# A Culturally Tailored, Behavioral Theory–Informed Educational Intervention for Pregnant Women to Improve Postnatal Care Use and Safe Maternal and Newborn Care Practices in Ethiopia: Protocol for a Cluster Randomized Controlled Trial

**DOI:** 10.2196/91271

**Published:** 2026-11-03

**Authors:** Firew Tiruneh Tiyare, Abraham Lomboro Dimore, Buzuneh Tasfa Marine, Manuel W Hetzel, Gunther Fink

**Affiliations:** 1 Swiss Tropical and Public Health Institute, Kreuzstrasse 2, Allschwil, Basel-Landschaft, 4123, Switzerland, 41 767722101; 2 University of Basel, Basel, Switzerland; 3 Jimma University, Jimma, Oromiya, Ethiopia

**Keywords:** health education, PNC use, Social Cognitive Theory, Theory of Planned Behavior, culturally tailored education, health-seeking behavior, Ethiopia, low- and middle-income countries, harmful cultural practice, newborn care

## Abstract

**Background:**

The postnatal period is a critical phase for maternal and newborn survival, during which many preventable complications and deaths occur, yet postnatal care (PNC) use remains low in Ethiopia. Care-seeking and postpartum practices are shaped by knowledge and also by interacting cognitive, interpersonal, sociocultural, and environmental determinants, including perceived risk, self-efficacy, family influence, social norms, and culturally embedded beliefs. Existing educational interventions largely prioritize information provision and insufficiently address these determinants, limiting the translation of knowledge into timely PNC use and safe maternal and newborn care practices.

**Objective:**

This study aims to evaluate the effectiveness of a culturally tailored educational intervention for pregnant women, informed by Social Cognitive Theory (SCT) and the Theory of Planned Behavior (TPB), in improving PNC use and promoting safe maternal and newborn care practices in Ethiopia.

**Methods:**

A 2-arm, assessor-blinded, open-label, matched-pair cluster randomized controlled trial was conducted across 14 woredas in Jimma Zone, Ethiopia. A total of 28 health facilities were randomly allocated in a 1:1 ratio to the intervention and control arms. Pregnant women at 20 to 24 weeks of gestation were recruited from kebeles within the randomized health-facility clusters. Participants in the intervention arm received the educational intervention delivered by trained health extension workers, whereas those in the control arm received routine antenatal health education. The primary outcome was PNC use; secondary outcomes were newborn-care knowledge and practices. Primary analyses will follow the intention-to-treat principle. Mixed-effects regression models with random intercepts for health-facility clusters will be used to estimate intervention effects while accounting for intracluster correlation.

**Results:**

The study was funded by the Eckenstein-Geigy Professorship in June 2025. From November 2025 to January 2026, 945 pregnant women were enrolled across 28 health facilities in Jimma Zone, Ethiopia. The intervention was delivered from December 2025 to May 2026, with follow-up conducted from December 2025 to July 2026. Follow-up data collection was completed in July 2026, and data cleaning and statistical analysis began in the same month. The primary and secondary analyses are scheduled for completion in fall 2026, and the trial findings are expected to be submitted for publication in fall 2026. The mean age of participants was 26.84 years (SD 5.23), with the majority residing in rural areas (n=834, 88.3%) and being married (n=937, 99.2%). Baseline characteristics were generally similar between trial arms regarding age, residence, marital status, and obstetric history, although notable differences were observed in maternal occupation, educational attainment, and household income.

**Conclusions:**

This trial will provide evidence on whether a culturally tailored, behavioral theory–informed educational intervention delivered during pregnancy can improve PNC use and promote safe maternal and newborn care practices in Ethiopia. The findings may inform the design and integration of context-responsive maternal and newborn health education within community-based health services.

## Introduction

The postnatal period, defined as the first 6 weeks after childbirth, is a critical phase in the maternal and newborn continuum of care and a central component of the World Health Organization (WHO) safe motherhood agenda [1]. It is during this period, particularly in the first hours and days after birth, that a substantial share of preventable maternal and neonatal mortality and morbidity occurs [2]. High-quality postnatal care (PNC) is central to improving maternal and newborn health because it promotes recommended care practices and facilitates the early identification, referral, and management of complications [3]. However, PNC continues to receive less attention than antenatal and intrapartum care in many low- and middle-income countries, including Ethiopia [4].

Although the definitions and timing of PNC vary across studies, available estimates consistently demonstrate substantial coverage gaps, particularly in sub-Saharan Africa [5]. Across 71 low- and middle-income countries, 76% of women were delivered in a health facility, but only 41% received a basic package of f4 delivery and immediate PNC [6]. In sub-Saharan Africa, an analysis of 36 countries estimated that 52.5% of women received at least one PNC contact, whereas a more recent study across 20 countries found that only 27.4% received adequate PNC [7,8]. Ethiopia shows a similar pattern of insufficiency. Although receipt of immediate PNC increased from 6.4% in 2011 to 33.4% in 2019, approximately two-thirds of women still did not receive care during the immediate postnatal period [9].

Low PNC use is particularly concerning because the postnatal period is one of the most vulnerable phases for mothers and newborns, yet much of their care shifts to the household, where decisions about care-seeking and newborn care practices are strongly influenced by family relationships, social norms, and culturally embedded beliefs about illness and protection [10]. While many traditional postpartum practices provide valuable emotional and practical support, others may unintentionally delay timely use of formal health services or increase health risks. For example, postpartum seclusion and concerns about spiritual harm may restrict mothers’ ability to seek timely care [11]. In addition, some newborns are given herbal preparations because they are believed to protect them from illness. These challenges are often more pronounced in rural settings, where limited health literacy, reduced access to reliable information and services, and greater reliance on customary practices can further reinforce such behaviors [12].

Addressing these challenges requires an understanding of the interacting cognitive, interpersonal, sociocultural, and environmental determinants that shape PNC use and postpartum practices [13,14]. The Theory of Planned Behavior (TPB) and Social Cognitive Theory (SCT) provide complementary frameworks for understanding and modifying these determinants [15,16]. TPB explains how attitudes, perceived social expectations, and perceived behavioral control shape women’s intentions to obtain PNC, with perceived control potentially influencing behavior directly. SCT complements this perspective by identifying the self-efficacy, outcome expectations, behavioral skills, observational learning, social support, and environmental conditions needed to translate intentions into action [17]. Integrating TPB and SCT is therefore appropriate for PNC research because postpartum care use is shaped by intentional, interpersonal, and contextual processes that no single individual-level model captures fully [18].

Despite this complexity, existing interventions to improve PNC use have largely relied on maternal education and information delivery [19]. Although these approaches have improved knowledge, some postpartum practices, and, in certain settings, PNC attendance, they have not consistently led to improved newborn care practices or completion of the recommended PNC contacts [20,21]. One possible explanation is that many interventions prioritize information provision while paying less attention to the cognitive, interpersonal, and contextual processes through which knowledge is translated into action [22]. For example, health education approaches that discourage established practices without exploring their underlying cultural meanings and perceived protective value are less likely to correct misconceptions or produce sustained behavior change and may instead be perceived as culturally incongruent [23,24].

Furthermore, many educational interventions provide limited opportunities for women to critically reflect on their beliefs, clarify personal values, and reconcile customary practices with evidence-based recommendations in a respectful, nonjudgmental manner [25,26]. They also rarely incorporate structured behavioral strategies, such as strengthening women’s self-efficacy to navigate household decision-making, manage social influences, and learn through credible community role models [27]. Consequently, interventions that are insufficiently responsive to local beliefs, family influences, and customary practices may have limited acceptability and behavioral effects [13]. This knowledge-behavior gap is also evident in Ethiopia, where reported exposure to PNC-related information during pregnancy coexists with potentially harmful postpartum practices and low completion of recommended PNC contacts [28].

Before intervention development, formative assessment involving pregnant and postpartum women, family members, and health care providers was conducted to understand the local determinants of PNC use and postpartum practices. The findings identified low perceived susceptibility to postpartum complications, limited recognition of maternal and newborn danger signs, postpartum seclusion, dependence on family approval and practical support, supernatural explanations of illness, reliance on traditional remedies, and misconceptions about recommended care. The research also revealed the protective intentions and deeply held values underlying some customary practices, culturally meaningful expressions used to encourage or discourage behaviors, and participants’ preference for interactive teaching using guided discussion, locally familiar scenarios, visual materials, and practical examples.

Based on the formative findings, the existing maternal health education approach was redesigned and culturally adapted in accordance with WHO PNC priorities, an integrated TPB–SCT framework, and the ADAPT guidance (Figure 1) [1,29-31]. This cluster randomized controlled trial (cRCT) primarily evaluates whether the redesigned maternal health education intervention, delivered during antenatal care (ANC), increases completion of at least 3 PNC contacts within 6 weeks after childbirth compared with routine maternal education. The secondary objectives are to assess its effects on knowledge of recommended newborn-care and cultural malpractices in Jimma Zone, Ethiopia.

**Figure 1.**
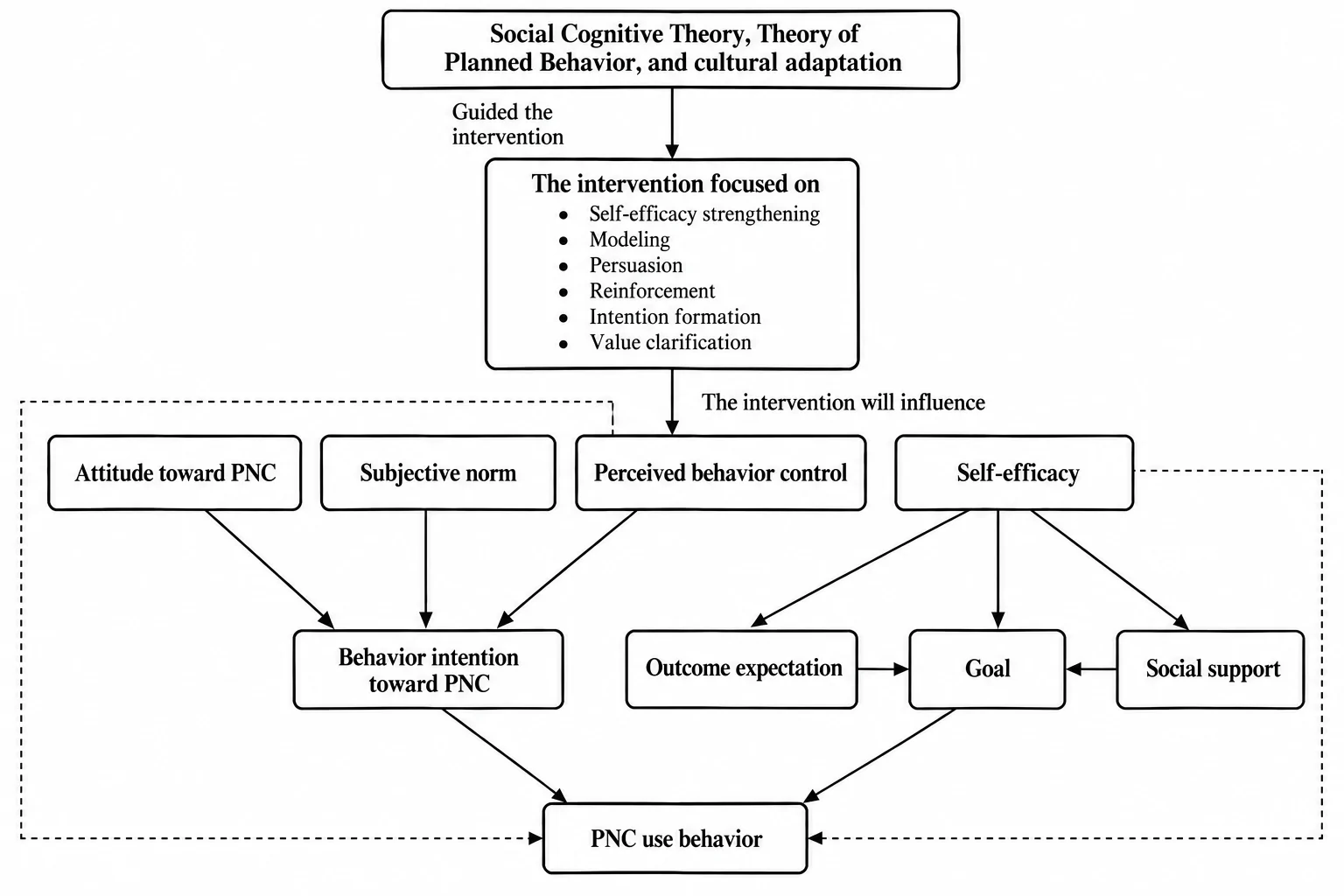
Conceptual framework of the culturally adapted, theory-informed educational intervention to improve postnatal care use (PNC).

## Methods

### Protocol Version

This manuscript describes protocol version 3.0, dated July 29, 2026. Important protocol modifications are documented through version-controlled records and communicated. The completed SPIRIT (Standard Protocol Items: Recommendations for Interventional Trials) checklist is provided as Checklist 1.

### Study Setting

The study will be conducted in Jimma Zone, which is located in the Oromia Regional State in southwestern Ethiopia, approximately 350 km from the capital city, Addis Ababa. Administratively, the Jimma Zone comprises 21 woredas (districts) and 2 town administrations, with an estimated population of 3.5 million. Most of the population resides in rural agrarian communities.

In the study area, health extension workers (HEWs) paid by the government serve as the frontline workforce at the community level, providing maternal and newborn health education, conducting routine home visits, mobilizing mothers through the Women’s Development Army, and facilitating referrals between households, health posts, and health centers. The HEWs also run “Pregnant Mothers’ Forums,” a community-based platform in which pregnant women gather at the health post for monthly group sessions led by HEWs and midwives. These sessions provide structured health education on a range of maternal and child health topics and serve as an important channel for counseling, peer support, and early engagement with essential maternal health services. These existing Pregnant Mothers’ Forums will serve as the primary platform for delivering the study intervention.

### Trial Governance and Oversight

The trial was coordinated by the principal investigator in collaboration with the research team at Jimma University, Ethiopia, and the Swiss Tropical and Public Health Institute (Swiss TPH), Switzerland, as well as the Jimma Zone Health Office and the participating woreda health offices in Jimma Zone, Ethiopia. The principal investigator was responsible for overall trial coordination, communication with participating sites, intervention implementation, regulatory compliance, and reporting. The supervisory team provided scientific and methodological oversight and reviewed trial progress. A local field-coordination team supervised participant recruitment, intervention delivery, follow-up, and adherence to the study protocol. The data-management team was responsible for database development, data-quality checks, secure data storage, and preparation of the analysis data set.

No end point adjudication committee was established because the primary and secondary outcomes were assessed using prespecified operational definitions and standardized data-collection procedures. No formal data monitoring committee was established because the intervention was educational and considered to present minimal risk. Trial conduct, participant safety, data quality, and intervention fidelity were instead monitored by the principal investigator and the supervisory team.

### Study Design

This study is designed as a 2-arm, assessor-blinded, open-label, parallel-group, cRCT. A total of 28 health facilities will serve as the units of randomization and will be allocated in a 1:1 ratio to the intervention and control groups (14 clusters per group).

### Sample Size

The study was designed to detect a 20% increase in PNC use, from an expected 28% in the control arm, based on the 2019 Ethiopia Mini Demographic and Health Survey [32]. The sample size calculation assumed an intracluster correlation coefficient of 0.10 and an average of 20 mothers per selected kebele. At randomization, 28 health-facility clusters were allocated equally to the intervention and control arms (14 clusters per arm). Within these clusters, kebeles served as the sampling units. One kebele was selected from each intervention cluster and 2 kebeles from each control cluster, resulting in 14 intervention kebeles and 28 control kebeles—a 1:2 intervention-to-control sampling ratio. With 20 women recruited from each of the 42 selected kebeles, the initial required sample size was 840 women. To account for an anticipated 15% (126/840) loss to follow-up, 3 additional women were added per kebele, resulting in a final target sample size of approximately 966 women. The unequal sampling ratio was adopted to improve statistical efficiency within the available budget, given the substantially higher cost of delivering the intervention than providing usual care.

### Study Population and Eligibility

The study population consists of pregnant women residing in the selected kebeles (the lowest administrative boundaries) of Jimma Zone, Oromia Regional State, Ethiopia, and meet the study’s eligibility criteria. The eligibility criteria guiding the selection of mothers were structured at 2 levels: cluster-level and participant-level eligibility. At the cluster level, health facilities were eligible if they served catchment populations of at least 5000 people, provided more than 50 ANC visits per month, and were located in periurban areas to ensure operational feasibility and reliable follow-up. At the participant level, eligibility was restricted to pregnant women between 20 and 24 weeks of gestation who resided within 1 hour of the selected health facility, thereby enabling timely and optimum exposure to the intervention and adequate follow-up until the postnatal period. Women were excluded if they planned to relocate outside the study area before completion of follow-up.

### Randomization and Concealment

Health facilities (health centers) will constitute the unit of randomization. Facilities will be randomized to the intervention or control arm using computer-generated random numbers, stratified by woreda, to ensure balance across administrative areas. The randomization sequence will be generated by the first author, who will not be involved in participant recruitment or outcome assessment. Allocation will be concealed until kebele selection and participant enrollment are completed. Outcomes will be measured at the individual level among mothers residing within the selected kebele catchment areas of the randomized facilities.

### Sampling Procedure

A multistage sampling approach will be used to select woredas, health facilities, kebeles, and study participants (Figure 2). First, in collaboration with the Zonal Health Department, 14 woredas were purposively selected based on safety, security, and logistical feasibility. All primary health care facilities within the selected woredas were subsequently screened using predefined criteria (catchment population >5000 and reliable accessibility). From the eligible facilities, 28 health facilities (2 per woreda) were selected using probability proportional to size and randomized, with the number of pregnant women in each facility’s catchment serving as the size measure.

**Figure 2.**
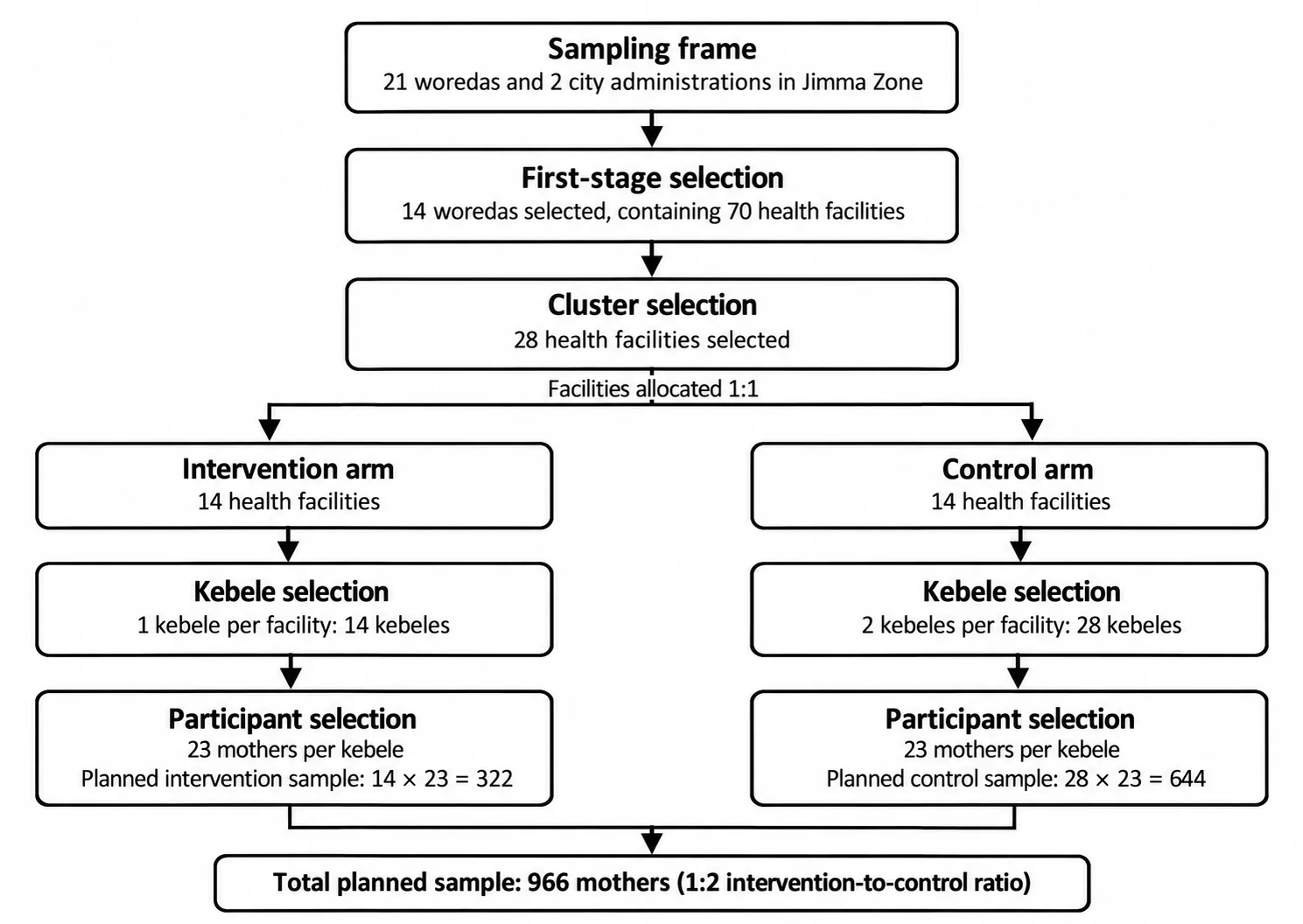
Sampling procedure and flow chart.

Subsequently, the 28 health facilities were grouped into 14 matched pairs according to woreda, with each pair comprising the 2 selected health facilities within the same woreda. Within each matched pair, 1 health facility was randomly allocated to the intervention arm and the other to the control arm. This matched-pair cluster randomization resulted in 14 health-facility clusters in each study arm. For each selected health facility, all kebeles with at least 23 eligible pregnant women were identified. One kebele was randomly selected from each intervention health-facility cluster, whereas 2 kebeles were randomly selected from each control health-facility cluster. Thus, 42 kebeles (14 in the intervention arm and 28 in the control arm) served as sampling units nested within the 28 randomized health-facility clusters. Each selected kebele inherited the treatment allocation of its corresponding health facility and was not randomized independently.

Finally, within each selected kebele, a sampling list of eligible pregnant women was compiled with support from HEWs and verified by a health extension supervisor to minimize coverage bias. From this list, a fixed number of 23 women per kebele were randomly selected using simple random sampling, producing a final target sample of 966 women across the 42 kebeles.

### Recruitment and Enrollment

Recruitment and enrollment will be carried out by trained data collectors who will verify eligibility, provide study information, and obtain informed consent. From the list of eligible women, 23 will be selected using simple random sampling, after receiving a detailed explanation of the study purpose, procedures, potential risks and benefits, voluntary participation, and confidentiality safeguards in their preferred local language. If a selected woman declines participation, a replacement will be randomly drawn from the remaining eligible list. Women who agree to participate will provide written or thumbprint-informed consent and will be enrolled immediately.

### Minimization of Bias

Several strategies will be implemented to minimize potential sources of bias. Randomization at the health-facility level will help balance both measured and unmeasured characteristics across groups, thereby reducing selection bias. Eligibility screening will be conducted using a standardized tool to ensure consistent application of inclusion and exclusion criteria. Data collectors will receive comprehensive training on interviewing techniques, nonleading questioning, and the use of standardized data collection instruments to minimize interviewer bias. To reduce information and recall bias, data will be collected prospectively at predetermined time points and linked with facility records whenever feasible. Data collectors will remain blinded to intervention allocation to the extent possible, particularly during outcome assessment. Additionally, recruitment and follow-up procedures will be closely supervised to ensure adherence to study protocols, enhance fidelity, and minimize variability across sites.

### Minimization of Loss to Follow-Up

Multiple approaches will be used to minimize loss to follow-up throughout the study period. Eligible women will be enrolled only if they reside within 1 hour of the study facility and do not anticipate moving outside the study area before completing the follow-up period. At enrollment, detailed locator information, including phone numbers, alternative contacts, and physical directions, will be recorded to facilitate timely tracking. Data collectors and HEWs will maintain scheduled home visits and reminder calls to ensure continuity of follow-up through pregnancy and the postnatal period. Engagement with community structures, including mothers’ forums and the Women’s Development Army, will further support retention by reinforcing communication and community-level follow-up. The study team will monitor follow-up completion weekly, and any missed visits will trigger immediate tracing efforts.

### The Conceptual Framework

The conceptual framework presents the hypothesized pathways through which the intervention is expected to influence PNC use. Specifically, the intervention is expected to strengthen attitudes toward PNC, subjective norms, perceived behavioral control, self-efficacy, outcome expectations, goal setting, social support, and behavioral intentions, ultimately promoting PNC use (Figure 1).

### Intervention Development and Validation

#### Intervention Overview

The intervention was developed as a redesigned maternal health education package that transforms routine information delivery into a structured, interactive, theory-informed, and context-responsive learning approach. The intervention included value clarification to influence behavioral beliefs, attitudes, and outcome expectations; guided discussion to address subjective norms and social support; problem-solving and action planning to strengthen perceived behavioral control and self-efficacy; and modeling, rehearsal, and supportive feedback to develop behavioral skills and confidence [33,34]. It is delivered during the antenatal period in community settings using a flipbook-based guide that supports HEWs and midwives in planning, organizing, and delivering standardized educational sessions. The guide specifies the learning objectives, session content, recommended duration and sequence, participatory learning activities, context-sensitive language, illustrative scenarios, practical examples, visual aids, and communication strategies used to facilitate mothers’ active participation.

#### Intervention Development

The intervention was developed through a systematic, theory-informed, and iterative process integrating evidence synthesis, formative assessment, behavioral theory, cultural adaptation, expert appraisal, pretesting, and feasibility piloting (Figure 3). First, the program context, available resources, and determinants of PNC use and newborn-care practices were examined. A review of the literature and relevant WHO recommendations identified recommended PNC and newborn-care content, documented barriers and facilitators, and potentially effective educational and behavior-change approaches. Formative assessment with women, family and community representatives, HEWs, and midwives provided additional insight into local beliefs, customary postpartum practices, family and community influences, service-related barriers, and preferred learning and communication approaches.

**Figure 3.**
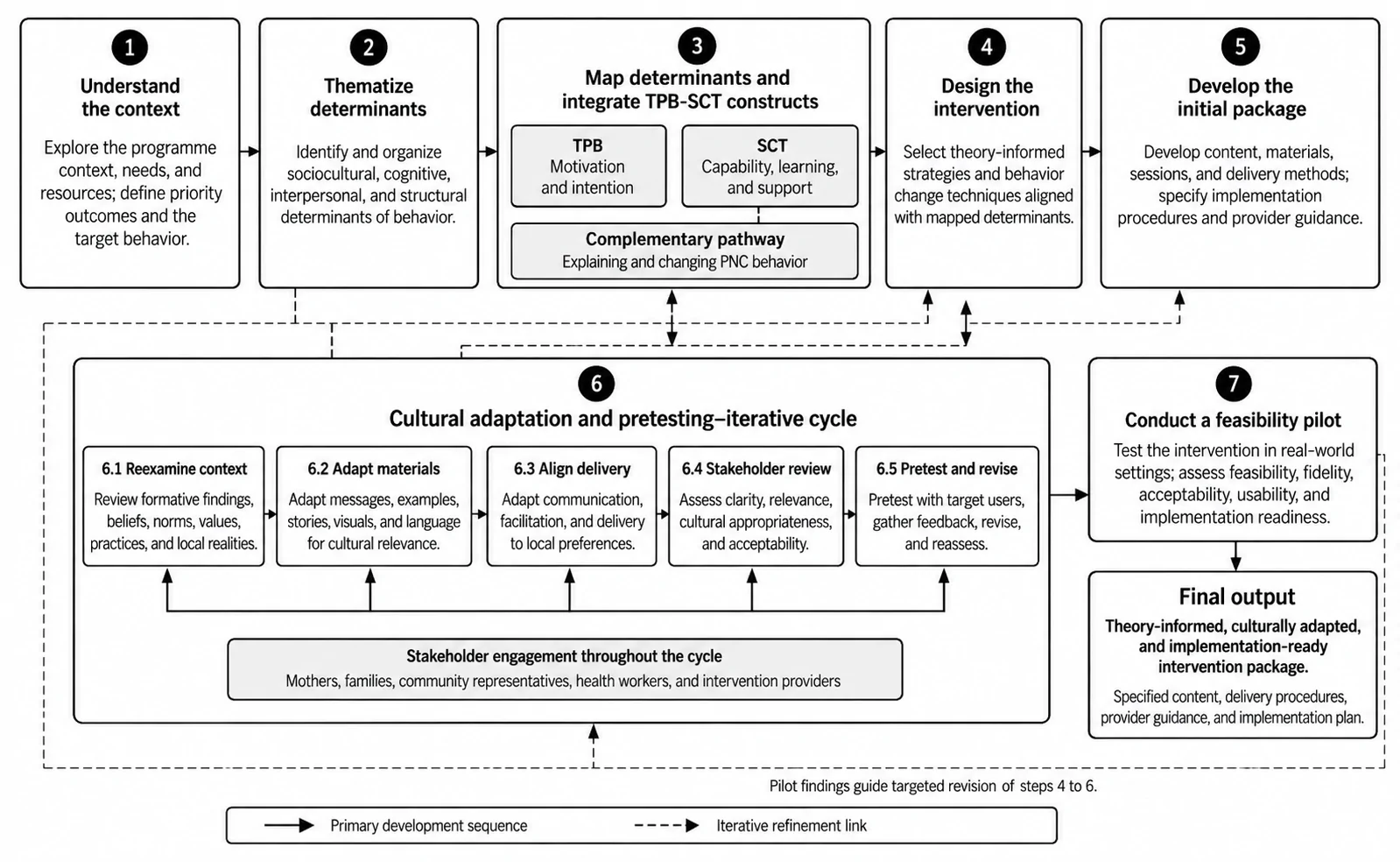
Schematic presentation of theory-informed culturally adapted intervention development framework. SCT: Social Cognitive Theory; TPB: Theory of Planned Behavior.

#### Theory Integration

Evidence from the literature review and formative assessment was analyzed using a combined inductive-deductive approach. Barriers and facilitators were first organized into informational, cognitive, interpersonal, sociocultural, and environmental determinants. These determinants were then mapped onto relevant constructs of the TPB and SCT [15,16], which provided complementary explanations of the motivational, social, and capability-related processes underlying PNC-seeking and newborn-care behaviors.

Beliefs about the benefits and consequences of PNC and customary postpartum practices were mapped onto TPB behavioral beliefs and attitudes and SCT outcome expectations. Perceived family expectations, community norms, and approval from influential individuals were linked to subjective norms and social influences. Women’s confidence in recognizing complications, communicating with family members, making care-seeking decisions, and overcoming barriers was mapped onto perceived behavioral control and self-efficacy. Opportunities to observe, discuss, and practice recommended behaviors were linked to observational learning and behavioral capability. Practical support, mobility restrictions, household decision-making, and access-related barriers were mapped onto SCT social and environmental influences and, where they affected women’s perceived ability to seek care, onto TPB perceived behavioral control.

This theoretical mapping informed the intervention objectives, key messages, and educational and behavior-change techniques selected for each determinant. Value-clarification and facilitated reflection activities addressed behavioral beliefs, attitudes, and outcome expectations. Guided discussions examined perceived social expectations and opportunities for family and community support. Problem-solving and action-planning activities targeted perceived behavioral control and self-efficacy, while modeling, rehearsal, and supportive feedback strengthened observational learning, behavioral capability, and confidence. Knowledge gaps and misconceptions were addressed through concise information, visual explanations, practical examples, and facilitated reflection rather than through information provision alone.

#### Intervention Design and Initial Package Development

Based on the identified determinants and their theoretical mapping, the intervention strategies, learning objectives, session content, and delivery methods were specified. The initial package comprised a structured flipbook, educational content, a facilitator guide, a provider-training curriculum, and an implementation manual. Together, these materials defined the sequence and duration of sessions, participatory teaching methods, key messages, illustrative scenarios, visual materials, communication guidance, provider responsibilities, and procedures for standardized implementation.

#### Cultural Adaptation and Iterative Refinement

Cultural adaptation was undertaken as an iterative process informed by the formative findings and established intervention-adaptation guidance [31]. Adaptation addressed both surface features—including language, illustrations, familiar examples, and presentation style—and deeper cultural dimensions, such as values, explanatory beliefs, protective intentions, family relationships, social expectations, and the meanings underlying customary postpartum practices. The adaptation cycle involved reexamining the formative context, adapting the materials, aligning delivery with local learning and communication preferences, obtaining stakeholder feedback, and pretesting and revising the package.

The intervention’s language, messages, scenarios, examples, visual materials, and communication strategies were adapted to reflect women’s lived experiences and the sociocultural context of the study setting. Local idioms and culturally meaningful expressions were incorporated, where appropriate, to reinforce recommended behaviors and respectfully encourage reflection on potentially harmful practices. Culturally rooted misconceptions were addressed without dismissing participants’ beliefs or the protective intentions underlying customary practices. Recommended behaviors were framed in ways that acknowledged existing family and community dynamics while strengthening women’s agency, confidence, and timely care-seeking.

Mothers, family and community representatives, HEWs, midwives, and other relevant stakeholders were engaged throughout the adaptation cycle. Their feedback informed the language, format, tone, sequencing, participatory methods, and delivery procedures, thereby improving the package’s cultural relevance, comprehensibility, acceptability, and feasibility.

#### Expert Appraisal, Pretesting, and Feasibility Piloting

The initial package was appraised by a multidisciplinary panel with expertise in maternal and newborn health, behavioral science, health education, public health, and community-based service delivery. The panel assessed scientific accuracy, consistency with WHO recommendations, alignment with TPB and SCT constructs, clarity, organization, cultural and linguistic appropriateness, and feasibility of delivery. Its recommendations were used to clarify the theoretical links, reorganize the sessions, and improve the educational and visual materials.

The revised package was then pretested with HEWs, midwives, and women who met the trial eligibility criteria but were not enrolled in the main trial. Pretesting examined the comprehensibility and acceptability of messages and visual materials, the cultural appropriateness of language and scenarios, the suitability of participatory activities, session duration and sequencing, and providers’ ability to use the flipbook and facilitator guide as intended.

A subsequent feasibility pilot assessed delivery under real-world conditions, including feasibility, fidelity, acceptability, usability, and implementation readiness. Feedback, observations, and pilot findings guided targeted revisions to the intervention content, cultural adaptations, delivery procedures, provider-training materials, and implementation guidance.

#### The Main Components and Contents

The intervention consisted of four structured educational sessions delivered monthly by trained HEWs and midwives through the existing Pregnant Mothers’ Forum platform. The educational content covered four core domains: (1) values and beliefs, focusing on clarifying maternal values and beliefs and addressing misconceptions; (2) social influences, addressing family objections and sociocultural norms surrounding postnatal care; (3) maternal and newborn health, emphasising the benefits of PNC, risks associated with missed care, and recommended maternal and newborn care practices; and (4) preparation for PNC, focusing on identifying individual barriers, setting goals, and developing practical plans for PNC attendance. Table 1 summarizes the timing, content, delivery methods, providers, educational materials, and targeted behavioral constructs for each session.

**Table 1.** Structure, content, delivery methods, and theory-informed behavioral targets of the intervention to improve postnatal care utilization and newborn care practices in Jimma Zone, Ethiopia.

Session	Timing and duration	Session content	Delivery methods	Provider and setting	Materials	Primary behavioral targets (TPB^a^/SCT^b^)
Session 1: value clarification and barriers to PNC^c^	Scheduled Pregnant Mothers’ Forum (ANC^d^); 15 to 20 min	Introduces PNC, engages value clarification, and identifies individual barriers to PNC use.	Interactive discussion and guided goal-setting exercises	Health extension workers (HEWs) and midwives; community Pregnant Mothers’ Forum	Standardized flipbook with visual illustrations	Attitudes (behavioral beliefs) and perceived behavioral control
Session 2: benefits of timely PNC	Scheduled Pregnant Mothers’ Forum (ANC); 15 to 20 min	Emphasizes the benefits of timely PNC and the risks associated with delaying or missing postnatal visits.	Role-modeling demonstrations, short educational videos, and facilitated discussion	HEWs and midwives; community Pregnant Mothers’ Forum	Flipbook, educational videos, and visual aids	Subjective Norms, self-efficacy, and observational learning
Session 3: essential maternal and newborn care	Scheduled Pregnant Mothers’ Forum (ANC); 15 to 20 min	Focuses on essential maternal and newborn care during the postnatal period, including maternal and newborn danger signs, breastfeeding support, thermal care, and maternal well-being.	Interactive discussion supported by practical examples and visual illustrations	HEWs and midwives; community Pregnant Mothers’ Forum	Flipbook and visual illustrations	Knowledge (behavioral capability) and self-efficacy
Session 4: review and readiness for PNC	Scheduled Pregnant Mothers’ Forum (ANC); 15 to 20 min	Summarizes key messages and guides participants through self-reflection and readiness assessment for timely PNC use.	Peer discussion and self-evaluation	HEWs and midwives; community Pregnant Mothers’ Forum	Flipbook	Behavioral Intention, self-efficacy, and perceived behavioral control

a TPB: Theory of Planned Behavior.

b SCT: Social Cognitive Theory.

c PNC: postnatal care.

d ANC: antenatal care.

### Study Procedures

The total planned duration of the study is 9 months, with recruitment begun in November 2025 and will continue for approximately 3 months. Data will be collected at 3 time points: baseline (20‐24 weeks of gestation), immediately after the intervention, and at 6 weeks postpartum, and the intervention will be implemented over a 4-month period. Each participant will be followed longitudinally from recruitment at 20 to 24 weeks of gestation until 6 weeks postpartum. Participants in the intervention arm will be invited to attend all intervention sessions.

Participants in the control clusters will continue to receive routine antenatal and maternal health education as part of the existing standard of care, delivered by HEWs and midwives during routine ANC and Pregnant Mothers’ Forums sessions. Routine education consists of unstructured counseling based on the national maternal and newborn health guidelines, covering general pregnancy, childbirth, and PNC messages, and is delivered according to usual practice without the structured flipbook, behavioral strategies, or theory-informed components of the intervention. To minimize contamination, intervention and control clusters are geographically separated, providers are trained only in the procedures assigned to their respective clusters, and intervention materials are not distributed outside intervention sites.

### Fidelity Monitoring

Fidelity monitoring will be implemented to ensure that the intervention is delivered as intended and to minimize variations in implementation across clusters. In the intervention kebeles, fidelity will be assessed using standardized checklists completed by supervisors, periodic observations of educational sessions, and brief participant feedback forms. These tools will document adherence to session content, delivery quality, participant engagement, and use of culturally adapted materials.

In the control kebeles, fidelity monitoring will focus on ensuring that routine care continues without exposure to intervention components. Supervisors will conduct brief periodic checks to verify that no intervention materials or messages are being delivered and that standard care practices remain unchanged. A simple checklist will be used to document compliance with routine care protocols.

### Risk Management and the Management of Adverse Events

The study team will implement procedures to identify and appropriately manage any maternal or newborn complications, including stillbirth, occurring during the study period. Although the intervention is educational and is not expected to increase clinical risks, participants may experience pregnancy-related complications as part of routine care. Any participant reporting or identified with complications will be referred promptly to the appropriate health facility for clinical assessment and management according to standard care protocols. The study team will document such events and report serious adverse events in accordance with ethical and regulatory requirements.

In the event of a stillbirth, the mother will receive appropriate support and counseling through the existing health system. Health workers involved in the study will provide compassionate communication, psychological first aid, and emotional support, and mothers requiring additional psychosocial support will be referred to available counseling services according to local practice and facility capacity. Participation in the study will not interfere with access to routine maternal and psychosocial care.

### Data Collection

Electronic data capture and computerized processing were used to improve data quality, reduce entry errors, and facilitate real-time monitoring of study indicators [35]. Trained data collectors, independent of intervention delivery, will administer all questionnaires using ODK Central. Baseline interviews will capture sociodemographic characteristics, obstetric history, newborn care knowledge, intention to seek PNC, behavioral determinants derived from TPB and SCT, and mobile phone access or prior exposure to PNC messages. During the intervention period, HEW and midwives will document session attendance, fidelity to session content, and participant engagement, with supervisors conducting periodic observations to ensure adherence to the protocol. Follow-up assessments at 6 weeks postpartum will measure PNC use, newborn care practices, newborn care knowledge, behavioral determinants, knowledge of maternal danger signs, and participant satisfaction.

### Outcome Measure

The primary outcome of PNC use, attendance at three or more PNC visits, will be measured using a structured maternal interview administered at the mother’s home at 6 weeks postpartum. Mothers will be asked whether they received a PNC check within 48 hours of birth and the total number of PNC contacts within 6 weeks. To minimize reporting bias, these responses will be verified against facility registers and maternal health cards issued during PNC visits.

The secondary outcome of newborn care knowledge will be assessed using a 22-item structured questionnaire (Multimedia Appendix 1) derived from the WHO PNC guidelines and previously published literature using a modified questionnaire developed by McConnell et al [36]. Items cover thermal care, breastfeeding, cord care, hygiene, and recognition of maternal and newborn danger signs. Responses are scored as correct/incorrect (Annex I); total scores will be summed and categorized into “good” vs “poor” knowledge based on predefined cutoffs.

### Data Management

This research project will implement a comprehensive data management plan. We will use digital data-capture and processing workflows to enable secure, automated handling and analysis of complex biophysical and survey data (eg, ODK with cloud backups and audit trails) [35]. All raw and processed data will be systematically collected, documented, and stored in secure, access-controlled repositories, in compliance with Swiss TPH guidelines. Metadata will be standardized following the FAIR (Findable, Accessible, Interoperable, and Reusable) data principles to facilitate future reuse and reproducibility. To prevent data loss due to hardware failure or human error, regular backups will be performed using both local and cloud-based storage solutions.

An audit trail will be maintained throughout the project to document all modifications made to the data, clearly recording when, how, and by whom changes were made, thereby enhancing transparency and reproducibility. A comprehensive data dictionary will accompany all data sets, providing clear definitions of variables, coding schemes, and units of measurement to ensure consistency across collaborators and future users. Data cleaning procedures will be conducted in Stata (version 18; StataCorp LLC) and fully documented using well-annotated do-files. Additionally, a logbook will be maintained in both digital and physical formats, as appropriate, to capture day-to-day research activities, data issues encountered, decisions made, and updates to protocols, further supporting the audit trail and ensuring full traceability of the research process.

Missing data will first be examined to determine their extent and patterns. If the proportion of missing data is minimal, complete case analyses will be performed. If missingness is substantial and considered compatible with a missing-at-random mechanism, multiple imputation will be considered as a sensitivity analysis.

### Statistical Analysis

All analyses will be conducted according to the intention-to-treat principle. Descriptive statistics will first be used to summarize participants’ baseline characteristics by study arm. Continuous variables will be presented as means and SDs or medians and IQR, while categorical variables will be summarized using frequencies and percentages. Baseline comparability between intervention and control groups will be assessed descriptively using standardized differences and appropriate statistical comparisons. Composite scores for newborn care knowledge and theoretical constructs, including attitudes, subjective norms, perceived behavioral control, behavioral intention, and self-efficacy, will be generated from multi-item measures. Internal consistency will be assessed using Cronbach α, and construct scores will be standardized (z scores) to improve comparability across domains.

The primary outcome, PNC use (coded as yes/no), will be analyzed using logistic regression models, while secondary outcomes, including maternal and newborn care knowledge scores, will be analyzed using logistic and linear regression models, respectively, according to the outcome distribution. Both unadjusted and adjusted models will be estimated. Adjusted models will include prespecified baseline covariates considered clinically or epidemiologically relevant or associated with the outcomes. Given the cluster-randomized design, all primary analyses will account for within-cluster correlation by using robust standard errors clustered at the health facility level. Intervention effects will be reported as odds ratios or regression coefficients with corresponding 95% CIs and 2-sided *P* values.

Mediation analysis will be conducted to assess whether changes in theory-based behavioral constructs mediate the effect of the intervention on PNC use and newborn care practices. Structural equation modeling or generalized structural equation modeling, depending on the distribution of the outcome variables, will be used to estimate the direct effect of the intervention on outcomes and the indirect effects operating through intermediate behavioral determinants. Indirect effects will be estimated using bootstrapped 95% CIs, and total, direct, and mediated effects will be reported.

Sensitivity analyses will be conducted to assess the robustness of intervention effect estimates under alternative modeling assumptions, including mixed-effects models, accounting for facility-level clustering, covariate-adjusted models using prespecified baseline characteristics, and alternative approaches to handling missing data, where appropriate.

### Ethical Considerations

Ethical approval was obtained from the Institutional Review Board of Jimma University, Ethiopia (approval number JUIH/IRB/0553/26), prior to study initiation. Additional ethical approval was granted by the Ethics Committee Northwest and Central Switzerland (EKNZ), Switzerland (approval number AO_ 2025‐00079) on September 28, 2026. The study protocol was further reviewed through the Swiss TPH Human Research Protection (HPR) process. The trial was prospectively registered at ClinicalTrials.gov (identifier: NCT07095296) before participant recruitment.

Written informed consent was obtained from all participants before enrollment. For participants younger than 18 years, written assent was obtained in addition to written consent from a parent or legal guardian, in accordance with Ethiopian ethical and legal requirements for research involving minors. Before consent was obtained, trained data collectors explained the study objectives, procedures, duration, potential risks and benefits, and participants’ rights, including the voluntary nature of participation and the right to withdraw at any time without affecting access to routine health services.

All study procedures were conducted in accordance with the ethical principles of the Declaration of Helsinki and applicable national and institutional regulations. Participant privacy and confidentiality were strictly maintained by assigning unique identification codes, removing personal identifiers from study data sets, storing study records securely with restricted access, and reporting findings only in aggregate form. No identifiable personal information or images will be disclosed.

## Results

### Trial Status and Progress

The study was funded by the Eckenstein-Geigy Professorship in June 2025. Participant recruitment was conducted from November 2025 to January 2026 across 28 health facilities in Jimma Zone, Ethiopia. All 28 health-facility clusters were randomized, with 14 allocated to the intervention arm and 14 to the control arm. A total of 945 pregnant women were enrolled.

The intervention was delivered from December 2025 to May 2026, and participant follow-up was conducted from December 2025 to July 2026. Follow-up data collection was completed in July 2026. Data cleaning and statistical analysis began in July 2026. The primary and secondary analyses are scheduled for completion in fall 2026, and the trial findings are expected to be submitted for publication in fall 2026.

The mean age of participants was 26.84 (SD 5.23) years, with the majority residing in rural areas (n=834, 88.3%) and being married (n=937, 99.2%). Baseline characteristics were generally similar between trial arms regarding age, residence, marital status, and obstetric history, although notable differences were observed in maternal occupation, educational attainment, and household income.

The intervention was delivered according to the prespecified protocol. Minor operational adaptations were made to participant scheduling and field implementation; however, these adaptations did not change the intervention content, cluster allocation, outcome definitions, or prespecified analytical procedures. No major protocol deviations affecting the conduct or integrity of the trial were identified.

### Baseline Characteristics

Baseline sociodemographic and obstetric characteristics were summarized descriptively for the overall study population and by trial arm (Table 2).

The key milestones and implementation timeline of the study, from funding and preparatory activities through recruitment, intervention delivery, follow-up, data analysis, and dissemination, are presented in (Table 3).

**Table 2.** Baseline sociodemographic and obstetric characteristics of participants by trial arm in Jimma Zone, Ethiopia (N=945)^a^.

Characteristics	Control (n=623)	Intervention (n=322)	Total (N=945)
Maternal age (y), mean (SD)	26.85 (5.28)	26.82 (5.14)	26.84 (5.23)
Maternal age group (y), n (%)			
<20	84 (13.5)	44 (13.7)	128 (13.5)
20‐29	350 (56.2)	182 (56.5)	532 (56.3)
≥30	189 (30.3)	96 (29.8)	285 (30.2)
Residence, n (%)			
Rural	546 (87.6)	288 (89.4)	834 (88.3)
Urban	77 (12.4)	34 (10.6)	111 (11.7)
Marital status, n (%)			
Married	616 (98.9)	321 (99.7)	937 (99.2)
Divorced	2 (0.3)	0 (0.0)	2 (0.2)
Widowed	2 (0.3)	1 (0.3)	3 (0.3)
Single	3 (0.5)	0 (0.0)	3 (0.3)
Religion, n (%)			
Muslim	570 (91.5)	306 (95.0)	876 (92.7)
Other	53 (8.5)	16 (5.0)	69 (7.3)
Maternal education, n (%)			
No formal education	215 (34.5)	91 (28.3)	306 (32.4)
Primary education	280 (44.9)	190 (59.0)	470 (49.7)
Secondary education or higher	128 (20.5)	41 (12.7)	169 (17.9)
Husband’s education, n (%)			
No formal education	210 (33.7)	92 (28.6)	302 (32.0)
Primary education	249 (40.0)	165 (51.2)	414 (43.8)
Secondary education or higher	164 (26.3)	65 (20.2)	229 (24.2)
Maternal occupation, n (%)			
Housewife	216 (34.7)	297 (92.2)	513 (54.3)
Employed	45 (7.2)	7 (2.2)	52 (5.5)
Farmer	299 (48.0)	6 (1.9)	305 (32.3)
Other	63 (10.1)	12 (3.7)	75 (7.9)
Household income quintile, n (%)			
Lowest	129 (20.7)	74 (23.0)	203 (21.5)
Low	111 (17.8)	64 (19.9)	175 (18.5)
Middle	162 (26.0)	114 (35.4)	276 (29.2)
High	70 (11.2)	35 (10.9)	105 (11.1)
Highest	151 (24.2)	35 (10.9)	186 (19.7)
Previous pregnancy, n (%)			
No	94 (15.1)	48 (14.9)	142 (15.0)
Yes	529 (84.9)	274 (85.1)	803 (85.0)
Antenatal care attendance, n (%)			
Did not attend	97 (15.6)	48 (14.9)	145 (15.3)
Attended	526 (84.4)	274 (85.1)	800 (84.7)
Pregnant Mothers’ Forum attendance, n (%)			
Did not attend	334 (53.6)	288 (89.4)	622 (65.8)
Attended	289 (46.4)	34 (10.6)	323 (34.2)

a Denominators may vary across variables because of missing data, including data not collected due to questionnaire skip logic. Percentages were calculated using the number of nonmissing observations for each variable.

**Table 3.** Study progress and timeline.

Study phase	Activity	Timeline	Status
Protocol development	Development of the protocol and intervention framework	December 2024-August 2025	Completed
Ethical approval	Approval from the relevant institutional review boards	October 2025	Completed
Trial registration	Registration of the cluster randomized trial	December 2025	Completed
Participant recruitment	Recruitment across 28 health facilities	November 2025-January 2026	Completed
Intervention delivery	Delivery of the educational intervention	December 2025-May 2026	Completed
Participant follow-up	Postnatal follow-up and outcome data collection	December 2026-July 2026	Completed
Data management	Data cleaning and quality checks	July 2026	Completed
Statistical analysis	Primary and secondary outcome analyses	August 2026- September 2026	Began in July 2026
Dissemination	Preparation and submission of the trial findings	October 2026-November 2026	Planned

## Discussion

This protocol describes a cRCT evaluating a culturally tailored, theory-driven educational intervention designed to improve PNC use and newborn care knowledge among pregnant women in Ethiopia. Grounded in the TPB and SCT [37,38], the intervention aims to strengthen both mothers’ intention and ability to seek timely PNC. We anticipate that participants receiving the culturally adapted, theory-based education will demonstrate higher newborn care knowledge, stronger intention to use PNC services, and greater adherence to recommended postpartum practices compared with those receiving standard care.

Efforts to improve PNC use are not new. Interventions such as SMS- and app-based messaging, video demonstrations, in-person counseling, and family-focused education have shown modest gains in PNC attendance and newborn care knowledge [39,40]. However, their effectiveness is often limited by several barriers, including low phone ownership, poor connectivity, and reliance on one-way information delivery with limited interactivity [41]. Crucially, increased knowledge does not consistently translate into sustained behavioral change when sociocultural norms and traditional postpartum practices remain unaddressed [13].

This study addresses these gaps by applying TPB and SCT within a unified framework rarely used in PNC education and by systematically adapting content using WHO guidance and local formative research. The intervention strengthens the capacity of HEWs and midwives to deliver culturally sensitive, behaviorally informed counseling through participatory communication, modeling, and structured goal setting. By equipping frontline providers with practical tools to address misconceptions and sociocultural barriers, the study seeks to foster meaningful and sustained improvements in PNC use and newborn care practices.

Evidence from prior interventions demonstrates the effectiveness of TPB- and SCT-based approaches in shaping health behaviors [42]. Studies show that TPB-based education improves knowledge, attitudes, and perceived behavioral control [43], while SCT-informed interventions have effectively supported behavior change in diverse health contexts [44]. Theory-driven strategies are better at bridging the gap between knowledge and sustained behavior change, especially when they include motivational and skills-building components [45], supporting the use of TPB and SCT to address the cultural, social, and psychological determinants of PNC-seeking in Ethiopia.

From a policy perspective, scalability and integration of culturally tailored, theory-based health education into routine antenatal and community health platforms are critical considerations. If effective, the findings will provide evidence that behaviorally informed, contextually grounded education delivered through existing community structures can meaningfully enhance PNC uptake in low-resource settings. Beyond PNC, this intervention may serve as a replicable model for applying behavioral science to enhance maternal health counseling in resource-constrained environments.

However, the study has several potential limitations. Blinding of data collectors and participants is not feasible, which may introduce performance or detection bias. Some risk of contamination across geographically proximate kebeles may persist despite facility-based randomization. Attrition may occur in highly mobile populations, and variation in provider motivation or workload could influence the fidelity of intervention delivery. Furthermore, reliance on maternal self-report for some outcomes may introduce recall bias.

To mitigate these concerns, multiple safeguards were incorporated into the protocol. Cluster randomization and clear separation of intervention materials minimize the risk of contamination. Intensive training, ongoing mentorship, and structured fidelity assessments aim to ensure consistent implementation. Comprehensive locator information, community engagement, and regular follow-up contacts are expected to reduce loss to follow-up. Where feasible, self-reported outcomes will be triangulated with facility records to improve data validity.

Overall, this trial is expected to generate robust evidence on the effectiveness of a culturally tailored, theory-driven educational intervention for improving PNC use in Ethiopia. Findings from this study have the potential to inform national policy, strengthen community-based maternal health strategies, and guide the scale-up of contextually adapted behavioral interventions in similar low-resource settings.

## Supplementary material

10.2196/91271 Multimedia Appendix 1 Newborn care knowledge and harmful practice questionnaire.

10.2196/91271 Checklist 1 SPIRIT checklist.[Author-notes equal-contrib1]

## Abbreviations

ANC: antenatal care
cRCT: cluster randomized controlled trial
EKNZ: Ethics Committee Northwest and Central Switzerland
FAIR: Findable, Accessible, Interoperable, and Reusable
HEW: Health extension worker
HPR: Human Research Protection
PNC: postnatal care
SCT: Social Cognitive Theory
SPIRIT: Standard Protocol Items: Recommendations for Interventional Trials
Swiss TPH: Swiss Tropical and Public Health Institute
TPB: Theory of Planned Behavior
WHO: World Health Organization
