# Supplementary material for: A Culturally Tailored, Behavioral Theory–Informed Educational Intervention for Pregnant Women to Improve Postnatal Care Use and Safe Maternal and Newborn Care Practices in Ethiopia: Protocol for a Cluster Randomized Controlled Trial
Source: JMIR Res Protoc. 2026 Nov 3;15:e91271. doi: 10.2196/91271 (PMC13730507; doi:10.2196/91271)
Supplement: Supplementary file 2 [file resprot-v15-e91271-s001.docx]

**Newborn Care Knowledge and harmful practice Questionnaire**

**Instrument details**

| **Field** | **Description** |
| --- | --- |
| **Module** | Newborn care knowledge |
| **Administration** | Interviewer-administered |
| **Response formats** | True/False, single response, and select all that apply |

**INTERVIEWER INSTRUCTION.** Read every question and response option exactly as written. Do not prompt, explain, or indicate whether an answer is correct. For ‘select all that apply’ questions, mark every response chosen by the participant.

**READ TO PARTICIPANT.** *Now I will ask you some questions about newborn care. Some statements are true and others are false. For each statement, please tell me whether you think it is true or false. For questions with several response options, please choose the answer or answers you think are correct.*

**BREASTFEEDING KNOWLEDGE**

**1.** Breastfeeding should be initiated within one hour after birth.

□ True	□ False

**2.** Colostrum (the first thick, yellowish breast milk) should be given to the newborn.

□ True	□ False

**3.** By three months of age, an infant can be given both breast milk and thin, sweetened porridge.

□ True	□ False

**4.** A mother should switch the baby to the other breast before the baby has finished feeding from the first breast.

□ True	□ False

**5.** Breastfeeding helps protect babies against illness.

□ True	□ False

**THERMAL CARE KNOWLEDGE**

**6.** A newborn should be bathed immediately after birth.

□ True	□ False

**7.** A newborn should be dried thoroughly immediately after birth to prevent heat loss.

□ True	□ False

**8.** A small or low-birthweight baby should be placed skin-to-skin on the mother's bare chest and covered to keep the baby warm.

□ True	□ False

**9.** A newborn's head should be covered with a clean, dry cap to reduce heat loss.

□ True	□ False

**UMBILICAL CORD CARE KNOWLEDGE**

**10.** Butter, petroleum jelly (Vaseline), herbs, or other substances should be applied to the umbilical cord stump.

□ True	□ False

**11.** The umbilical cord stump should be tightly covered with a cloth or bandage.

□ True	□ False

**12.** The umbilical cord should be cut with a clean, sterile instrument.

□ True	□ False

**13.** For a home birth, a clean, sterile cord tie or clamp should be prepared before delivery.

□ True	□ False

**KNOWLEDGE OF NEWBORN DANGER SIGNS**

**14.** Which of the following are newborn danger signs that require the baby to be taken immediately to a health facility? Select all that apply.

□ The baby is unable to breastfeed or has stopped feeding well.

□ The baby feels unusually hot to the touch.

□ The baby has convulsions.

□ There is redness, swelling, or pus around the umbilical cord.

□ The palms or soles are yellow.

□ The baby has difficulty breathing or is breathing unusually fast.

□ The baby feels unusually cold to the touch.

**EYE CARE KNOWLEDGE**

**15.** Breast milk, herbs, or other non-prescribed substances can be put into a newborn's eyes to prevent infection.

□ True	□ False

**16.** Which of the following are appropriate practices when cleaning a newborn's eyes? Select all that apply.

□ Wash hands with soap and clean water before and after cleaning the baby's eyes.

□ If cleaning is needed, use clean water and a clean, soft cloth or cotton.

□ Wipe each eye gently, using a separate clean part of the cloth or a separate piece of cotton for each eye.

**SUN-SAFETY KNOWLEDGE**

**17.** When, if ever, should a newborn be intentionally exposed to direct sunlight? Select one response.

□ A newborn should not be intentionally exposed to direct sunlight; keep the baby in the shade when outdoors.

□ Early morning.

□ Midday.

□ Afternoon.

□ Evening.

**18.** If a newborn is taken outdoors during daylight, how should the baby be protected? Select one response.

□ Keep the baby in the shade and use light protective clothing, including a cap.

□ Wrap the baby completely in heavy clothing.

□ Leave the baby completely uncovered.

□ Expose the baby directly to sunlight with only the eyes and genital area covered.

**19.** Routine direct sunlight exposure is necessary for a newborn's health.

□ True	□ False

**HAND-HYGIENE KNOWLEDGE**

**20.** When should a caregiver wash their hands with soap and clean water to protect a newborn? Select all that apply.

□ Before touching or handling the baby.

□ After changing the baby's diaper or cleaning stool or urine.

□ Before breastfeeding or feeding the baby.

□ After using the toilet.

**Harmful practices**

**21.** Mothers in Jimma use various practices to keep their newborns safe and healthy. Some practices may be beneficial, while others may be harmful. Which, if any, of the following practices did you use for your newborn? (Select all that apply)

- Applied butter, oil, petroleum jelly (Vaseline), ash, or cow dung to the umbilical cord stump
- Gave the newborn water, butter, or herbal medicine before initiating breastfeeding
- Discarded the first breast milk (colostrum)
- Gave the newborn abish (fenugreek) or another substance to promote weight gain
- Had traditional uvulectomy performed on the newborn
- Massaged the newborn’s abdomen
- Moulded or pressed the newborn’s head to change its shape
- None of the above
- Other (specify): __________

# Scoring guide

Award one point for each correct response unless otherwise noted; award zero points for an incorrect or unselected response.

| **Item** | **Correct response** | **Scoring or interpretation** |
| --- | --- | --- |
| 1. | True | Breastfeeding should begin within one hour after birth. |
| 2. | True | Colostrum should be given. |
| 3. | False | Exclusive breastfeeding is recommended for the first six months. |
| 4. | False | Allow the baby to finish the first breast before offering the other. |
| 5. | True | Breastfeeding helps protect against childhood illness. |
| 6. | False | Delay the first bath for at least 24 hours. |
| 7. | True | Dry the newborn thoroughly immediately after birth. |
| 8. | True | Skin-to-skin care helps maintain warmth. |
| 9. | True | A clean, dry cap reduces heat loss. |
| 10. | False | Keep the cord clean and dry; do not apply routine substances. |
| 11. | False | Do not tightly cover the cord stump. |
| 12. | True | Use a clean, sterile cutting instrument. |
| 13. | True | Prepare a clean, sterile cord tie or clamp before a home birth. |
| 14. | All seven options | One point for each danger sign recognized; maximum 7 points. |
| 15. | False | Do not place breast milk, herbs, or non-prescribed substances in the eyes. |
| 16. | All three options | One point for each appropriate practice selected; maximum 3 points. |
| 17. | No intentional direct sunlight | Report with a context-specific sun-safety domain. |
| 18. | Shade and protective clothing | Report with a context-specific domain. |
| 19. | False | Routine direct sunlight exposure is not necessary and should not be encouraged. |
| 20. | All four options | One point for each correct handwashing occasion; maximum 4 points. |
| 21. | No options are correct | A women who did not select any option has no harmful practices |

The total score will be standardized using the sample mean and standard deviation. Participants with a z score of 0 or higher—corresponding to a score at or above the sample mean—will be classified as having “good knowledge,” whereas those with a z score below 0 will be classified as having “poor knowledge.”

## References

1. World Health Organization. Breastfeeding: initiation within one hour and exclusive breastfeeding for six months. https://www.who.int/health-topics/breastfeeding

2. World Health Organization. Essential newborn care. https://www.who.int/teams/maternal-newborn-child-adolescent-health-and-ageing/newborn-health/essential-newborn-care

3. World Health Organization. Caring for a newborn: drying, delayed bathing, thermal care, dry cord care, and hand hygiene. https://www.who.int/tools/your-life-your-health/life-phase/newborns-and-children-under-5-years/caring-for-newborns

4. World Health Organization. Newborn mortality: essential care and newborn danger signs. https://www.who.int/news-room/fact-sheets/detail/newborn-mortality

5. World Health Organization. Vitamin D supplementation for infants: limiting ultraviolet exposure and avoiding direct sunlight. https://www.who.int/tools/elena/bbc/vitamind-infants
